# HER2 status and disparities in luminal breast cancers

**DOI:** 10.1002/cam4.757

**Published:** 2016-06-01

**Authors:** Andreana N. Holowatyj, Julie J. Ruterbusch, Manohar Ratnam, David H. Gorski, Michele L. Cote

**Affiliations:** ^1^ Department of OncologyWayne State University School of MedicineDetroitMichigan; ^2^ Population Studies and Disparities ProgramBarbara Ann Karmanos Cancer InstituteDetroitMichigan; ^3^ Molecular Therapeutics ProgramBarbara Ann Karmanos Cancer InstituteDetroitMichigan; ^4^ Michael and Marian Ilitch Department of SurgeryWayne State University School of MedicineDetroitMichigan

**Keywords:** Breast cancer, disparities, HER2, hormone receptor, luminal, SEER

## Abstract

National Comprehensive Care Network guidelines for adjuvant treatment of invasive breast cancer are based on HER2 and hormone receptor (HR) status, where HR+ disease encompasses all estrogen receptor (ER)+ and/or progesterone receptor (PR)+ tumors. We sought to explore clinical and demographic differences among patients with HR+ breast cancer subtypes, and the role of HER2 status, age, race/ethnicity, and socioeconomic status (SES) in disease risk. We evaluated breast cancer subtype distribution, defined by HR and HER2 status, using patient clinical, demographic, and socioeconomic characteristics. Differences in HR categories by demographic and tumor characteristics were examined using chi‐squared tests. Multinomial logistic regression was used to estimate odds ratios (OR) and 95% confidence intervals (CI) to quantify associations between breast cancer HR status and demographic factors. We found that differences in HR+ (ER−/PR+ vs. ER+/PR− or ER+/PR+) tumor biology are likely clinically significant and may play a role in breast cancer, regardless of HER2 status. While clinical and patient characteristics differed within each luminal subtype, we found disparities in SES only among Luminal A (HR+/HER2−) tumors. Among HR+/HER2− cases, we observed that ER−/PR+ patients tend to live in areas of higher poverty (OR = 1.20, 95% CI = 1.03–1.40) and are 70% more likely to be aged 50 years or older. However, this pattern was not found in women with Luminal B (HR+/HER2+) disease (Poverty OR = 0.98, 95% CI = 0.76–1.27; Age OR = 1.01, 95% CI = 0.81–1.26). Racial/ethnic disparities among non‐Hispanic black and Hispanic women persisted across HR+/HER2− cases compared to non‐Hispanic white women. Our findings suggest that while race/ethnicity and SES are correlated, each plays an independent role in contributing to disease among Luminal A tumors. Further study is needed to investigate how tumor biology, race/ethnicity, and socioeconomic disparities among HR+/HER2− cases may contribute to poorer patient prognosis.

## Introduction

Breast cancer is the most common cancer diagnosed in women in the United States, accounting for 29% of newly diagnosed female cancers. An estimated 246,660 new cases and 40,450 deaths are anticipated from the disease in 2016 1. Breast cancer most commonly arises from the mammary ductal epithelium, and its systemic treatment is guided by its molecular characteristics. Specifically, National Comprehensive Care Network (NCCN) guidelines for the treatment of invasive breast cancer outline systemic adjuvant therapies based on hormone receptor (HR) and human epidermal growth factor receptor 2 (HER2) statuses, where positive HR status is defined as expression of the estrogen (ER) and/or progesterone (PR) receptors 2. Clinically, tumor cells are evaluated for these biological markers to approximate cancer molecular subtype based on expression profiling (Luminal A/B, HER2−enriched, basal‐like).

Luminal breast cancers account for about 60% of all cases. They are HR‐positive (HR+) and can be further classified based on HER2 receptor status 3. Luminal A breast cancers are HER2−negative (HR+/HER2−) and include ER+/PR+, ER+/PR−, and ER−/PR+ status. Their adjuvant treatment includes endocrine therapy with or without multimodality chemotherapy, based on tumor size, lymph node status, and, more recently, the 21‐gene recurrence score 2. In contrast, Luminal B tumors tend to be more aggressive, demonstrate HER2−enrichment (HR+/HER2+), and encompass ER+/PR+, ER+/PR−, and ER−/PR+ cases. Recommended treatment for Luminal B tumors includes anthracycline‐based trastuzumab‐containing multimodality chemotherapy, followed by a 1‐year course of trastuzumab and 5 years of endocrine therapy 2. Together, luminal breast cancer subtypes are associated with the best short‐term prognoses for patients, attributable to favorable responses to hormonal therapy 4, 5.

Across breast cancer subtypes, there exist demographic and socioeconomic status (SES) differences 6. Previous reports have found that differences in SES may reflect underlying differences in exposures to known breast cancer risk factors, as women with higher SES tended to have lower parity and more frequent use of exogenous hormones 7, 8, 9. Additionally, SES has been found to interact with race/ethnicity among female breast cancers 10. SES has previously been associated with negative HR status (HER2−enriched and basal‐like subtypes) in breast cancer 7, 11, 12. SES disparities have also persisted in HR+ subtypes (HR+/HER2− and HR+/HER2+) 13. We therefore sought to explore further clinical and demographic differences in order to identify disparities among female patients with luminal (HR+) breast cancer subtypes using Surveillance, Epidemiology, and End Results (SEER) data by assessing estrogen and progesterone receptor status, and the role of SES in risk of disease.

## Methods

### Data sources and case selection

Data were obtained from the National Cancer Institute's SEER program. The SEER program collects cancer incidence and mortality data from 20 population‐based cancer registries covering approximately 28% of the US population 14. SEER*Stat is a free program provided by SEER to access and analyze information in the publicly available database. A case listing session in SEER*Stat was run on the SEER 18 incidence dataset to obtain demographic, tumor characteristics, and socioeconomic information on breast cancers 15. The SEER 18 incidence dataset includes information from the following registries: Alaska Native Tumor Registry, Arizona Indians, Cherokee Nation, Connecticut, Detroit, Atlanta, Greater Georgia, Rural Georgia, San Francisco‐Oakland, San Jose‐Monterey, Greater California, Hawaii, Iowa, Kentucky, Los Angeles, Louisiana, New Jersey, New Mexico, Seattle‐Puget Sound, and Utah. The dataset used is publicly available and was exempt from human subjects review. Therefore, an IRB protocol was not required for this study.

Women with invasive breast cancer diagnosed from 2010 to 2012 were included in this study. Diagnosis year 2010 is the first year for which HER2 status data were available. Study data, including ER, PR, and HER2 status, demographic characteristics, and tumor stage, were identified across SEER registries using standardized coding rules based on pathology reports and hospital medical records. Case patients diagnosed with nevi and melanomas; soft tissue tumors and sarcomas, NOS; fibromatous neoplasms; lipomatous neoplasms; myomatous neoplasms; fibroepithelial neoplasms; synovial‐like neoplasms; blood vessel tumors; osseous and chondromatous neoplasms; miscellaneous bone tumors; gliomas; nerve sheath tumors; and granular cell tumors and alveolar soft part sarcoma histologies were excluded. To assess breast cancer subtype, tumors were classified into six mutually exclusive categories: ER+/PR+/HER2−; ER+/PR−/HER2−; ER−/PR+/HER2−; ER+/PR+/HER2+; ER+/PR−/HER2+; ER−/PR+/HER2+. We restricted our analyses to exclude patients without positive/negative ER, PR, or HER2 statuses (*n* = 19,020). Case patients with ER−/PR−/HER2+ (HER2−enriched) and ER−/PR−/HER2− (Basal‐like) receptor status were also excluded (*n* = 26,736). The final analytic dataset consisted of 134,639 breast cancer patients.

The variables of interest included age at diagnosis (<50, 50–64, 65–74, 75+ years), race/ethnicity (non‐Hispanic (NH) white, NH black, NH Asian, or Pacific Islander, NH American Indian, Hispanic), American Joint Commission of Cancer (AJCC) clinical stage, and tumor size (≤0.5 cm, >0.5 cm). An approximation of SES was evaluated using contextual measures of area‐based poverty. The percentage of persons and families whose incomes were below 200% of the poverty level were calculated in SEER using county attribute data from the US Census Bureau's 2008–2012 American Community Survey. We categorized our cohort of breast cancer cases into quartiles based on the percentage of persons with incomes below 200% of the poverty level distribution within the dataset. Quartile 1 (Q1) (<24%) represented the fourth of the cohort that reside in areas where less than 24% of residents are from low‐income category. We defined quartile 2 (Q2) (24% ≤ 31% low income), quartile 3 (Q3) (31% ≤ 39%), and quartile 4 (Q4) (>39%), such that Q4 contained the fourth of our cohort that reside in areas with the highest proportion of low‐income residents. Follow‐up for each case was current within 22 months of the annual submission date (1 November 2014).

### Statistical analysis

Pair‐wise comparisons of hormone receptor (HR) categories by demographic and tumor characteristics were examined using chi‐squared tests. Multinomial logistic regression was used to estimate odds ratios (OR) and 95% confidence intervals (CI) to quantify associations between breast cancer hormone receptor status and various demographic factors. Analyses were stratified by HER2 status and the reference outcome category was ER+/PR+ disease. Demographics variables assessed, included: age at diagnosis (<50, 50–64, 65–74, 75+), race/ethnicity (NH white, NH black, NH Asian or Pacific Islander, NH American Indian, Hispanic), and area‐based poverty level (quartiles). These factors were adjusted for age, race/ethnicity, and poverty level based on patients having complete information for each of these covariables. All data were analyzed using SAS version 9.4 statistical software (SAS Institute; Cary, NC). All statistical tests were two‐sided. A *P *< 0.05 was considered to be statistically significant.

## Results

### Differences among ER+/PR+, ER+/PR−, and ER−/PR+ hormone receptor‐positive breast cancers

Expression of the estrogen (ER) and/or progesterone (PR) receptors was defined as hormone receptor‐positive (HR+) disease 2. To study the clinical, demographic, and socioeconomic differences within Luminal A (HR+/HER2−) and Luminal B (HR+/HER2+) clinical breast cancer subtypes, we gathered 134,639 patients with HR+ and known HER2 receptor status from the SEER database 6. Among these patients, 118,285 (87.8%) cases were HR+/HER2− (Luminal A) (Table 1) and 16,354 (12.2%) were HR+/HER2+ (Luminal B) tumors (Table 2).

**Table 1:** Patient and tumor characteristics of Luminal A invasive breast cancer cases

	*N*	Luminal A (HER2−)						*P*‐value^a^		
		ER+/PR+		ER+/PR−		ER−/PR+		ER+/PR+:	ER+/PR+:	ER+/PR−:
		*N*	%	*N*	%	*N*	%	ER+/PR−	ER−/PR+	ER−/PR+
Total	118,285	102,087		14,994		1204				
Age at diagnosis								<0.0001	<0.0001	<0.0001
<50	21,777	19,299	18.9	2081	13.9	397	33.0			
50–64	43,550	37,261	36.5	5849	39.0	440	36.5			
65–74	28,705	24,836	24.3	3670	24.5	199	16.5			
≥75	24,253	20,691	20.3	3394	22.6	168	14.0			
Race/ethnicity								<0.0001	<0.0001	<0.0001
NH white	85,717	74,701	73.2	10,297	68.7	719	59.7			
NH black	10,540	8371	8.2	1930	12.9	239	19.9			
Asian/Pacific Islander	9117	7956	7.8	1094	7.3	67	5.6			
Am.Indian/AlaskaNative	633	555	0.5	71	0.5	7	0.6			
Hispanic	11,429	9766	9.6	1496	10.0	167	13.9			
Unknown	849	738	0.7	106	0.7	5	0.4			
AJCC stage								<0.0001	<0.0001	<0.0001
0–I	64,491	57,029	55.9	7018	46.8	444	36.9			
II	34,905	29,601	29.0	4809	32.1	495	41.1			
III	11,352	9329	9.1	1868	12.5	155	12.9			
IV	4979	3997	3.9	900	6.0	82	6.8			
Unknown	2558	2131	2.1	399	2.7	28	2.3			
Tumor size								0.1503	<0.0001	<0.0001
≤0.5 cm	10,091	8724	8.5	1316	8.8	51	4.2			
>0.5 cm	104,379	90,257	88.4	13,021	86.8	1101	91.4			
Unknown	3815	3106	3.0	657	4.4	52	4.3			
Poverty index								0.0833	<0.0001	<0.0001
Q1	28,333	24,551	24.0	3516	23.4	266	22.1			
Q2	27,526	23,778	23.3	3513	23.4	235	19.5			
Q3	28,566	24,569	24.1	3736	24.9	261	21.7			
Q4	33,837	29,170	28.6	4226	28.2	441	36.6			
Unknown	23	19	0.0	3	0.0	1	0.1			

Summary of clinical and demographic characteristics of hormone receptor‐positive (HR+), HER2− Luminal A breast cancers in women with invasive breast cancer: Surveillance, Epidemiology and End Results 18, 2010–2012. NH, non‐Hispanic; ER, estrogen receptor; PR, progesterone receptor; HER2, human epidermal growth factor receptor 2.

a *P*‐value calculations do not include unknown values.

**Table 2:** Patient and tumor characteristics of Luminal B invasive breast cancer cases

	*N*	Luminal B (HER2+)						*P*‐value^a^		
		ER+/PR+		ER+/PR−		ER−/PR+		ER+/PR+:	ER+/PR+:	ER+/PR−:
		*N*	%	*N*	%	*N*	%	ER+/PR−	ER−/PR+	ER−/PR+
Total	16,354	11,391		4491		472				
Age at diagnosis								<0.0001	0.7842	<0.0001
<50	4621	3518	30.9	950	21.2	153	32.4			
50–64	6631	4431	38.9	2016	44.9	184	39.0			
65–74	2886	1951	17.1	855	19.0	80	16.9			
≥75	2216	1491	13.1	670	14.9	55	11.7			
Race/ethnicity								0.3661	<0.0001	<0.0001
NH white	10,793	7545	66.2	2970	66.1	278	58.9			
NH black	1918	1306	11.5	555	12.4	57	12.1			
Asian/Pacific Islander	1491	1038	9.1	400	8.9	53	11.2			
Am. Indian/Alaska Native	105	63	0.6	30	0.7	12	2.5			
Hispanic	1921	1352	11.9	503	11.2	66	14.0			
Unknown	126	87	0.8	33	0.7	6	1.3			
AJCC stage								<0.0001	<0.0001	0.003
0–I	6383	4534	39.8	1710	37.9	139	29.2			
II	5792	4073	35.8	1536	34.2	183	38.8			
III	2463	1697	14.9	676	15.1	90	19.1			
IV	1260	781	6.9	437	9.7	42	8.9			
Unknown	456	306	2.7	132	2.9	18	3.8			
Tumor size								<0.0001	0.1152	0.7848
≤0.5 cm	1348	861	7.6	443	9.9	44	9.3			
>0.5 cm	14,178	9983	87.6	3800	84.6	395	83.7			
Unknown	828	547	4.8	248	5.5	33	7.0			
Poverty index								0.1446	0.6807	0.3295
Q1	3648	2514	22.1	1025	22.8	109	23.1			
Q2	3717	2585	22.7	1030	22.9	102	21.6			
Q3	3814	2635	23.1	1078	24.0	101	21.4			
Q4	5172	3654	32.1	1358	30.2	160	33.9			
Unknown	3	3	0.0	0	0.0	0	0.0			

Summary of clinical and demographic characteristics of hormone receptor‐positive (HR+), HER2+ Luminal B breast cancers in women with invasive breast cancer: Surveillance, Epidemiology and End Results 18, 2010–2012. NH, non‐Hispanic; ER, estrogen receptor; PR, progesterone receptor; HER2, human epidermal growth factor receptor 2.

a *P*‐value calculations do not include unknown values.

For Luminal A (HR+/HER2−) cases, 102,087 (86.3%) were ER+/PR+; 14,994 (12.7%) ER+/PR−; and 1204 (1.0%) ER−/PR+ (Table 1). Luminal A subtype patients demonstrated significantly different distributions by age, race/ethnicity, tumor size, AJCC clinical stage, and SES measures of poverty (*P* < 0.0001). Specifically, Luminal A cases with ER−/PR+ status were more likely to be diagnosed at a younger age, to be NH black or Hispanic, to live in counties with higher poverty, to have larger tumors, and to present with later stage disease (Table 1).

Of the Luminal B (HR+/HER2+) tumors, 11,391 (69.7%) were ER+/PR+; 4491 (27.4%) ER+/PR−; and 472 (2.9%) ER−/PR+ (Table 2). Patients with Luminal B breast cancers had distributions that varied by age, race/ethnicity, and AJCC clinical stage. Compared with ER+/PR− and ER+/PR+ patients, Luminal B cases with ER−/PR+ status were more likely to be diagnosed at a younger age, to be NH Asian, Pacific Islander, or Hispanic, and to be diagnosed at later stages of disease (Table 2). Among Luminal B tumors, we observed no significant differences in SES, measured with county‐level poverty (*P* = 0.1446, *P* = 0.6807, and *P* = 0.3295).

An additional 19,020 breast cancer patients had borderline or unknown status for ER, PR, or HER2, and were not included in this analysis. Among these women, 67.7% were NH white (12,876 cases), 11.7% were NH black (2227 cases), 10.9% were Hispanic (2068 cases), 7.4% were Asian or Pacific Islander (1404 cases), 0.5% were American Indian/Alaska Native (91 cases), and 1.9% (354 cases) had unknown race/ethnicity (data not shown). Eighty three percent of unknown cases were diagnosed with invasive breast cancer at age 50 years and older (15,793 cases). 61.5% of cases were diagnosed with early stage (AJCC stage 0–II) disease (11,701 cases), 17.8% were diagnosed with later stage (AJCC stage III–IV) cancer, and 20.1% (3929 cases) did not have information on AJCC stage. 59.1% of cases (11,242 cases) resided in areas where at least 31% of residents were living in poverty (Q3 or Q4). Together, cases with unknown receptor status tended to be NH white, older, and diagnosed with early‐stage disease (data not shown).

Overall, we observed that, regardless of HER2 status, ER−/PR+ cases were more likely to be diagnosed in young patients (age <50 years) and to present with later stage (stage III–IV) disease, but were less likely to be NH white, as compared to ER+/PR− or ER+/PR+ patients.

### Socioeconomic disparities in Luminal A breast cancers

We also noted significant differences in area‐based poverty among patients with Luminal A (HR+/HER2−) tumors. To further explore the relationship of SES and HR+ status (ER+/PR+, ER+/PR−, or ER−/PR+) within each breast cancer subtype, we used multinomial logistic regression models. Because race and age are associated with SES, we adjusted for these variables to determine whether socioeconomic disparities persisted in this population.

Table 3 summarizes results from models adjusted for area‐based poverty, age, and race/ethnicity. Using ER+/PR+ tumors as the referent outcome in each subtype and poverty quartile Q1 as the referent covariable, we found that women with Luminal A breast cancer who live in counties with higher poverty were more likely to be diagnosed with ER−/PR+ disease (Q4: OR = 1.20, 95% CI = 1.03–1.40) (Table 3). Women diagnosed with ER−/PR+ Luminal A disease were 1.7‐fold more likely to be under 50 years of age compared to women with ER+/PR+ tumors in our area‐based poverty‐adjusted models. In addition, NH blacks were at an increased risk of being diagnosed with ER−/PR+ Luminal A breast cancers (OR = 2.62, 95% CI = 2.25–3.05). Notably, age, race, and poverty were not associated with ER−/PR+ disease in Luminal B breast cancers (Table 3).

**Table 3:** Adjusted odds ratios (OR) for patient demographics and socioeconomic status by hormone receptor‐positive (HR+) breast cancers

	Area‐based poverty^a^			
	Luminal A (HER2−)^b^		Luminal B (HER2+)^c^	
	ER+/PR−OR (95% CI)	ER−/PR+OR (95% CI)	ER+/PR−OR (95% CI)	ER−/PR+OR (95% CI)
Age at diagnosis				
<50	0.67 (0.64–0.71)	1.68 (1.47–1.93)	0.59 (0.54–0.65)	1.01 (0.81–1.26)
50–64^d^	1	1	1	1
65–74	0.96 (0.91–1.00)	0.70 (0.59–0.82)	0.97 (0.88–1.07)	0.99 (0.75–1.29)
≥75	1.08 (1.03–1.13)	0.72 (0.60–0.86)	1.01 (0.90–1.12)	0.94 (0.69–1.28)
Race/ethnicity				
NH white^d^	1	1	1	1
NH black	1.75 (1.66–1.85)	2.62 (2.25–3.05)	1.14 (1.02–1.28)	1.18 (0.88–1.58)
Asian/Pacific Islander	1.05 (0.98–1.12)	0.77 (0.59–0.99)	1.03 (0.91–1.17)	1.36 (1.00–1.84)
Am. Indian/Alaska Native	0.96 (0.75–1.23)	1.22 (0.57–2.57)	1.27 (0.82–1.97)	5.15 (2.74–9.67)
Hispanic	1.19 (1.12–1.26)	1.48 (1.25–1.76)	1.04 (0.93–1.16)	1.31 (0.99–1.73)
Poverty index				
Q1^d^	1	1	1	1
Q2	1.02 (0.97–1.07)	0.89 (0.75–1.07)	0.96 (0.87–1.07)	0.90 (0.68–1.19)
Q3	1.01 (0.96–1.06)	0.88 (0.74–1.05)	0.97 (0.88–1.08)	0.88 (0.66–1.16)
Q4	0.93 (0.89–0.98)	1.20 (1.03–1.40)	0.88 (0.79–0.97)	0.98 (0.76–1.27)

NH, non‐Hispanic; ER, estrogen receptor; PR, progesterone receptor; HER2, human epidermal growth factor receptor 2.

a Model adjusted for age, race, and poverty (quartiles).

b Referent group is HER2−/ER+/PR+.

c Referent group is HER2+/ER+/PR+.

d Referent covariable.

## Discussion

In this study, we explored associations among luminal breast cancers and SES as assessed by age, race/ethnicity, and a measurement of county‐level poverty and found that within Luminal A (HR+/HER2−) and Luminal B (HR+/HER2+) cancers, clinical and demographic characteristics varied. Clinical differences among luminal breast cancers can be attributed to the opposing effects of estrogen and progesterone on tumor progression. Estrogen supports tumor growth but suppresses progression, whereas, progesterone supports tumor progression and is associated with more aggressive disease 16, 17. In the absence of estrogen signaling (ER− tumors), high progesterone levels in women have been shown to support tumor progression without opposition from estrogen 18, 19. Consistent with these findings, we observed in this study that regardless of HER2 status, women with ER−/PR+ tumors were more likely to present with later stage (stage III–IV) disease compared to ER+/PR+ or ER+/PR− cases. These results suggest that differences in HR+ (ER−/PR+ vs. ER+/PR− or ER+/PR+) tumor biology are likely to be clinically significant and play a role in breast cancer disease, regardless of HER2 status.

Demographic characteristics of patients, including age, also varied within each luminal breast cancer subtype. Progesterone levels are higher in premenopausal women, typically those diagnosed with breast cancer under the age of 50 years, compared to postmenopausal women over the age of 60 20, 21. Our study showed that women under the age of 50 were at an increased risk of developing ER−/PR+ Luminal A disease, while women over the age of 60 were at a decreased risk compared to ER+/PR+ disease. This observation is consistent with reports that high progesterone levels (occurring only in the luteal phase and in pregnancy) will induce breast cancer cell invasiveness and metastasis in the absence of estrogen or the ER. In contrast, age was not associated with increased risk of ER−/PR+ disease among Luminal B cases. This observation may be explained by findings using experimental models, that overexpression of HER2 supports aggressive tumor growth in luminal breast cancers 22.

Racial and ethnic differences were also noted among Luminal A cancers, as NH black women were most likely to develop ER−/PR+ disease. Indeed, previous studies have demonstrated that among Luminal A tumors, race‐associated biological factors contribute to poorer outcomes in black women compared to NH white women 23, 24. Among women with Luminal B tumors, NH Asian or Pacific Islander or American Indian/Alaska Native individuals were at an increased risk of developing ER−/PR+ tumors. However, caution should be taken in interpreting results in these racial/ethnic categories due to small sample size. Earlier findings have observed that Hispanic white women are more likely to present with more aggressive tumors and be diagnosed with ER+/PR− disease 25. In our study, we confirmed that Hispanic individuals were at an increased risk of developing ER+/PR− or ER−/PR+ disease compared to ER+/PR+ status in both Luminal A and B breast cancer subtypes after adjusting for age, race, and area‐based poverty.

While clinical and patient characteristics differed within each luminal breast cancer subtype, we found disparities in SES persist only among Luminal A tumors. Luminal A breast cancers are associated with the most favorable short‐term prognosis due to favorable responses to endocrine therapy 4, 5. However, assessment of long‐term prognosis demonstrates similar or worse overall survival for Luminal A cases as compared to other subtypes 26. In our study, among Luminal A cases, we observed that ER−/PR+ disease was associated with residing in areas of higher poverty even after adjusting for age and race/ethnicity. Caution should be exercised in the interpretation for results of ER−/PR+ cases due to small sample size. The relationship between SES and development of Luminal A breast cancers demonstrates that while race and SES are correlated, each plays an independent role in contributing to disease among Luminal A tumors. These disparities were not observed among Luminal B tumors, suggesting that HER2 status may be associated with risk factors that affect the SES of patients.

Our study is the first to assess disparities among HR‐positive (HR+) breast cancer subtypes in the context of HER2 status using SEER patient data. Previous studies have investigated the role of HR+ status without HER2 information, or have analyzed the role by breast cancer subtype. For example, a recent study by Parise et al. that used the California Cancer Registry found that SES moderately altered racial disparities and risk of mortality in particular breast cancer subtypes 27. Similar results have been observed among other tumor sites 10. While our findings suggest differences within Luminal A or B breast cancer subtypes, available data for HER2 are limited to 3 years of diagnosis, which does not allow further analysis for SES disparities in mortality at this time. The use of data from the population‐based SEER program is a strength of this study, as it allows for the inclusion of a considerable number of pathologically verified cases making our results more generalizable to the larger United States population. Another strength is that these data are of high quality and database entries are standardized and continuously monitored for accuracy. However, use of SEER data does have limitations. Family history, lifestyle‐related factors (e.g., obesity, reproductive factors, and environmental exposures), modality of diagnosis, and chemotherapy data, are not available for study. We were also unable to examine patients whose ER, PR, or HER2 status was unknown.

Scientific, clinical, and public health implications can be inferred from this study. First, our findings are consistent with preclinical and clinical data regarding the opposing effects of the estrogen and progesterone receptors in breast cancer growth and progression. Further research is needed to analyze the opposing effects of hormone receptors in the context of HER2 status. Hormone receptor‐positive (HR+) breast cancers are currently defined as ER and/or PR‐positive tumors. In addition, our observations suggest that additional work is required to assess clinical differences we observed, particularly between ER−/PR+ and ER+/PR− breast cancers, as this information could potentially be used to improve systemic adjuvant treatments. Disparities in SES among Luminal A (HR+/HER2−) breast cancer patients may be associated with risks of recurrence and mortality, and identifying barriers in patient access to medical care can seek to improve patient outcomes in underserved, high‐risk populations. Overall, detailed investigations of differences in tumor biology and association of race/ethnicity and SES among HR‐positive breast cancers, particularly those with HER2−negative status (HR+/HER2−), may lead to the identification of additional prognostic markers, direct resources to underserved populations for screening, and improve adjuvant treatments to better long‐term patient outcomes.

## Conflict of Interest

The authors declare that they have no conflicts of interest.

## References

[1] Siegel, R. L. , K. D. Miller , and A. Jemal . 2016 Cancer statistics, 2016. CA Cancer J. Clin. 66:7–30. 26742998 10.3322/caac.21332

[2] National Comprehensive Care Network . 2015 Breast cancer. NCCN Clinical Practice Guidelines in Oncology. 10.6004/jnccn.2004.0021 19795607

[3] Perou, C. M. , A. L. Borresen‐Dale . 2011 Systems biology and genomics of breast cancer. Cold Spring Harb. Perspect. Biol. 3:a003293. 21047916 10.1101/cshperspect.a003293 PMC3039533

[4] Voduc, K. D. , M. C. Cheang , S. Tyldesley , K. Gelmon , T. O. Nielsen , and H. Kennecke . 2010 Breast cancer subtypes and the risk of local and regional relapse. J. Clin. Oncol. 28:1684–1691. 20194857 10.1200/JCO.2009.24.9284

[5] Rastelli, F. , and S. Crispino . 2008 Factors predictive of response to hormone therapy in breast cancer. Tumori 94:370–383. 18705406 10.1177/030089160809400314

[6] Howlader, N. , S. F. Altekruse , C. I. Li , V. W. Chen , C. A. Clarke , L. A. Ries , et al. 2014 US incidence of breast cancer subtypes defined by joint hormone receptor and HER2 status. J. Natl. Cancer Inst. 106:dju055. doi: 10.1093/jnci/dju055. 24777111 10.1093/jnci/dju055 PMC4580552

[7] Akinyemiju, T. F. , M. Pisu , J. W. Waterbor , and S. F. Altekruse . 2015 Socioeconomic status and incidence of breast cancer by hormone receptor subtype. Springerplus 4:508. 26405628 10.1186/s40064-015-1282-2 PMC4573746

[8] Palmer, J. R. , E. Viscidi , M. A. Troester , C. C. Hong , P. Schedin , T. N. Bethea , et al. 2014 Parity, lactation, and breast cancer subtypes in African American women: results from the AMBER Consortium. J. Natl. Cancer Inst. 106:dju237. doi: 10.1093/jnci/dju237. 25224496 10.1093/jnci/dju237 PMC4271113

[9] Heck, K. E. , and E. R. Pamuk . 1997 Explaining the relation between education and postmenopausal breast cancer. Am. J. Epidemiol. 145:366–372. 9054241 10.1093/oxfordjournals.aje.a009114

[10] Kish, J. K. , M. Yu , A. Percy‐Laurry , and S. F. Altekruse . 2014 Racial and ethnic disparities in cancer survival by neighborhood socioeconomic status in surveillance, epidemiology, and end results (SEER) registries. J. Natl. Cancer Inst. Monogr. 2014:236–243. 25417237 10.1093/jncimonographs/lgu020 PMC4841168

[11] Andaya, A. A. , L. Enewold , M. J. Horner , I. Jatoi , C. D. Shriver , and K. Zhu . 2012 Socioeconomic disparities and breast cancer hormone receptor status. Cancer Causes Control 23:951–958. 22527173 10.1007/s10552-012-9966-1

[12] Dunn, B. K. , T. Agurs‐Collins , D. Browne , R. Lubet , and K. A. Johnson . 2010 Health disparities in breast cancer: biology meets socioeconomic status. Breast Cancer Res. Treat. 121:281–292. 20437200 10.1007/s10549-010-0827-x

[13] Sineshaw, H. M. , M. Gaudet , E. M. Ward , W. D. Flanders , C. Desantis , C. C. Lin , et al. 2014 Association of race/ethnicity, socioeconomic status, and breast cancer subtypes in the National Cancer Data Base (2010‐2011). Breast Cancer Res. Treat. 145:753–763. 24794028 10.1007/s10549-014-2976-9

[14] Howlader, N. , M. Krapcho , J. Garshell , D. Miller , S. F. Altekruse , C. L. Kosary , et al., eds. 2015 SEER cancer statistics review 1975–2012. National Cancer Institute, Bethesda, MD Available at: http://seer.cancer.gov/csr/1975_2012/. Based on November 2014 SEER data submission, posted to the SEER web site, April 2015.

[15] Surveillance Epidemiology and End Results (SEER) Program (www.seer.cancer.gov). SEER*Stat Database: Incidence ‐ SEER 18 Regs Research Data + Hurricane Katrina Impacted Louisiana Cases, Nov 2014 Sub (1973‐2012 varying) ‐ Linked To County Attributes ‐ Total U.S., 1969‐2011 Counties, National Cancer Institute, DCCPS, Surveillance Research Program, Surveillance Systems Branch. released April 2015, based on the November 2014 submission.

[16] McFall, T. , M. Patki , R. Rosati , and M. Ratnam . 2015 Role of the short isoform of the progesterone receptor in breast cancer cell invasiveness at estrogen and progesterone levels in the pre‐ and post‐menopausal ranges. Oncotarget 6:33146–33164. 26356672 10.18632/oncotarget.5082 PMC4741755

[17] Wang, X. , K. Belguise , N. Kersual , K. H. Kirsch , N. D. Mineva , F. Galtier , et al. 2007 Oestrogen signalling inhibits invasive phenotype by repressing RelB and its target BCL2. Nat. Cell Biol. 9:470–478. 17369819 10.1038/ncb1559 PMC2394707

[18] Kariagina, A. , J. Xie , I. M. Langohr , R. C. Opreanu , M. D. Basson , and S. Z. Haslam . 2013 Progesterone decreases levels of the adhesion protein E‐cadherin and promotes invasiveness of steroid receptor positive breast cancers. Horm. Cancer 4:371–380. 23996076 10.1007/s12672-013-0158-6 PMC10358015

[19] Lin, V. C. , A. S. Eng , N. E. Hen , E. H. Ng , and S. H. Chowdhury . 2001 Effect of progesterone on the invasive properties and tumor growth of progesterone receptor‐transfected breast cancer cells MDA‐MB‐231. Clin. Cancer Res. 7:2880–2886. 11555606

[20] Geisler, J. 2003 Breast cancer tissue estrogens and their manipulation with aromatase inhibitors and inactivators. J. Steroid Biochem. Mol. Biol. 86:245–253. 14623518 10.1016/s0960-0760(03)00364-9

[21] Pasqualini, J. R. , G. Chetrite , C. Blacker , M. C. Feinstein , L. Delalonde , M. Talbi , et al. 1996 Concentrations of estrone, estradiol, and estrone sulfate and evaluation of sulfatase and aromatase activities in pre‐ and postmenopausal breast cancer patients. J. Clin. Endocrinol. Metab. 81:1460–1464. 8636351 10.1210/jcem.81.4.8636351

[22] Ithimakin, S. , K. C. Day , F. Malik , Q. Zen , S. J. Dawsey , T. F. Bersano‐Begey , et al. 2013 HER2 drives luminal breast cancer stem cells in the absence of HER2 amplification: implications for efficacy of adjuvant trastuzumab. Cancer Res. 73:1635–1646. 23442322 10.1158/0008-5472.CAN-12-3349 PMC3600586

[23] D'Arcy, M. , J. Fleming , W. R. Robinson , E. L. Kirk , C. M. Perou , and M. A. Troester . 2015 Race‐associated biological differences among Luminal A breast tumors. Breast Cancer Res. Treat. 152:437–448. 26109344 10.1007/s10549-015-3474-4 PMC4527078

[24] O'Brien, K. M. , S. R. Cole , C. K. Tse , C. M. Perou , L. A. Carey , W. D. Foulkes , et al. 2010 Intrinsic breast tumor subtypes, race, and long‐term survival in the Carolina Breast Cancer study. Clin. Cancer Res. 16:6100–6110. 21169259 10.1158/1078-0432.CCR-10-1533 PMC3029098

[25] Banegas, M. P. , and C. I. Li . 2012 Breast cancer characteristics and outcomes among Hispanic black and Hispanic white women. Breast Cancer Res. Treat. 134:1297–1304. 22772379 10.1007/s10549-012-2142-1 PMC3903405

[26] Blows, F. M. , K. E. Driver , M. K. Schmidt , A. Broeks , F. E. van Leeuwen , J. Wesseling , et al. 2010 Subtyping of breast cancer by immunohistochemistry to investigate a relationship between subtype and short and long term survival: a collaborative analysis of data for 10,159 cases from 12 studies. PLoS Med. 7:e1000279. 20520800 10.1371/journal.pmed.1000279 PMC2876119

[27] Parise, C. A. , and V. Caggiano . 2015 The influence of socioeconomic status on racial/ethnic disparities among the ER/PR/HER2 breast cancer subtypes. J. Cancer Epidemiol. 2015:813456. 26339244 10.1155/2015/813456 PMC4539118

